# Execution-Aware Segmented Modeling of Temporally Correlated Flux-Induced Phase Noise in Quantum Circuits

**DOI:** 10.3390/e28080845

**Published:** 2026-07-29

**Authors:** Hongxiang Zhu, Xinxuan Chen, Hui-Hai Zhao, Feng Wu, Zhaofeng Su

**Affiliations:** 1 School of Computer Science and Technology, University of Science and Technology of China, Hefei 230026, China; zhxustc@mail.ustc.edu.cn (H.Z.); xinxuanchen@mail.ustc.edu.cn (X.C.); 2 Zhongguancun Laboratory, Beijing 100000, China; zhaohuihai@iqubit.org; 3 Research Institute of Quantum Technology, The Hong Kong Polytechnic University, Hong Kong

**Keywords:** superconducting quantum computing, temporally correlated noise, flux-induced phase noise, circuit-level simulation, noise modeling, randomized benchmarking, dynamical decoupling

## Abstract

Temporally correlated flux-induced phase noise can influence superconducting-quantum-circuit execution in ways that are not fully captured by uncorrelated, memoryless, or gate-averaged noise models. In this work, we develop an execution-oriented, circuit-level workflow for modeling and evaluating such effects. The workflow combines source-specific circuit-level noise components with a phenomenological segmented correlation-time construction for flux-induced phase noise, thereby enabling explicit control of a tunable correlation-time parameter $\tau_{c}$ within a composite circuit-level noise model. Using a single-qubit Carr–Purcell–Meiboom–Gill (CPMG) sequence and standard randomized benchmarking as the representative single-qubit circuit settings, we evaluate how circuit outputs respond to temporally correlated flux-induced phase noise under otherwise matched simulation conditions. The results show that temporally correlated flux-induced phase noise produces circuit-level behavior that differs qualitatively from uncorrelated or memoryless descriptions, and that its impact is governed jointly by the correlation-time parameter $\tau_{c}$ , the temporal structure of the circuit, and the way in which the circuit samples the noise. The proposed workflow provides a circuit-level framework for analyzing temporally correlated noise in superconducting quantum computing.

## 1. Introduction

Meaningful evaluation of quantum-circuit performance requires error models whose implications can still be interpreted at the level of circuit execution, rather than only through isolated component metrics or average gate characteristics. Superconducting qubits provide a particularly important setting for this issue, as they have become one of the leading platforms for quantum information processing and are now routinely studied in regimes of increasing processor size and circuit depth [1,2]. In such settings, circuit outputs are shaped not only by the strength of individual error channels, but also by how those errors are encountered, accumulated, and exposed over the course of execution. Circuit-level noise modeling therefore becomes a necessary part of performance evaluation when the objective is to understand execution outcomes beyond isolated error characterization.

This execution-level perspective is also relevant to quantum error correction, where repeated syndrome-extraction circuits involve idling, entangling operations, measurement, reset, and schedule-dependent fault propagation [3,4,5]. In such settings, logical performance cannot be inferred reliably from isolated gate metrics alone, because physically structured noise must be embedded into the circuit-execution process itself in order to understand how it is accumulated, sampled, and ultimately reflected in logical-level performance [4,5]. Circuit-level noise modeling is therefore valuable not only for the analysis of individual circuits, but also as a methodological basis for studying error-correction-oriented workloads in superconducting quantum computing. In the present work, single-qubit CPMG and randomized benchmarking circuits are used as controlled diagnostic testbeds to isolate how temporal-memory effects interact with pulse timing and circuit duration. This controlled setting establishes an execution-level modeling and analysis step that can be extended in subsequent work to multiqubit schedules, syndrome-extraction circuits, and QEC-oriented workloads.

Many circuit-level studies represent noise through independent, memoryless, or gate-averaged channels, which provide a practical abstraction for scalable simulation and evaluation [6]. These approximations, however, may suppress distinctions that become important when the underlying noise contains substantial low-frequency content or nontrivial temporal structure. In superconducting qubits, low-frequency noise constitutes an important class of error sources associated with decoherence and control errors [7,8]. Among these low-frequency noise mechanisms, flux noise is particularly relevant in the present context, as it has been linked to frequency fluctuations, dephasing, and gate-dependent phase accumulation [9]. Moreover, noise-spectroscopy studies based on dynamical decoupling and driven evolution have shown that the effect of such noise cannot always be reduced to a single static error scale, because its spectral and temporal structure can influence how errors are expressed over experimentally relevant execution times [9,10].

Existing studies have already shown that temporally structured noise in superconducting qubits can be characterized and analyzed beyond purely static error descriptions. Non-Markovian processes have been experimentally reconstructed and partially controlled on superconducting quantum processors [11], and experimentally informed reconstructions have been used to build predictive descriptions of non-Markovian behavior in superconducting qubits [12]. Related work has also examined signatures of non-Markovianity in effective superconducting-qubit models [13], while correlated noise mechanisms in multiqubit superconducting devices have been connected to directly observable error behavior [14]. Complementary circuit-level studies have also highlighted the importance of execution-aware modeling for structured error mechanisms in superconducting processors; for example, SurgeQ examined how coupling-strength tuning and crosstalk-aware scheduling jointly affect large-scale circuit fidelity under a composite noise setting [15]. More directly related to the present study, O’Brien et al. [3] represented low-frequency flux noise through a phase offset that remains quasi-static within each circuit run and is applied to flux-sensitive operations. This whole-run quasi-static representation provides the circuit-level starting point for the segmented extension considered here. Together, these studies make clear that temporal memory and correlated structure are not peripheral effects. Complementary approaches infer temporal structure from measured noise spectra through filter-function analysis [9], or describe environmental memory through microscopic system–environment dynamics, for example, by using real-time path-integral methods [16]; the present work instead focuses on a compact execution-level representation of the effective phase-covariance structure relevant to circuit execution. What remains less directly addressed, however, is a circuit-level workflow in which flux-induced phase noise is embedded into the circuit-execution timeline through an explicit and tunable segmented temporal scale, enabling controlled comparisons under otherwise matched execution settings.

To address this limitation, we develop an execution-oriented, circuit-level workflow for modeling and evaluating temporally structured flux-induced phase noise in superconducting quantum circuits. The workflow links sampled phase offsets to the circuit-execution timeline through a segmented representation parameterized by the temporal scale $\tau_{c}$ , and incorporates this construction into a broader composite-noise setting that also includes idling noise, single-qubit control noise, and photon-induced dephasing. This design allows us to examine how temporally structured flux-induced phase noise is expressed at the level of circuit execution under otherwise matched simulation conditions, and to identify circuit-level temporal-memory effects that differ qualitatively from uncorrelated or memoryless noise descriptions. The segmented construction is used as a controlled phenomenological representation of finite temporal memory at the execution level and provides a basis for subsequent calibration to device-specific noise statistics.

The contributions of this work are threefold.

1. We develop a phenomenological, execution-oriented, segmented correlation-time model for representing flux-induced phase noise at the circuit level, with explicit control of the temporal scale $\tau_{c}$ .
2. We use this framework to show that the circuit-level response to temporally correlated flux-induced phase noise is governed jointly by the correlation-time parameter $\tau_{c}$ , the temporal structure of the circuit, and the way in which the circuit samples the noise.
3. We demonstrate these effects on two complementary classes of single-qubit circuits, namely a structured Carr–Purcell–Meiboom–Gill (CPMG) sequence and standard randomized benchmarking, thereby revealing both the circuit-dependent onset of effectively slow-noise behavior and the progressive deviation of RB decay from the standard single-exponential picture.

The remainder of this paper is organized as follows: Section 2 presents the modeling workflow and the segmented correlation-time construction for flux-induced phase noise. Section 3 describes the experimental setup, including noise settings, circuits, and evaluation metrics. Section 4 reports the main results. Section 5 concludes the paper and outlines possible future directions.

## 2. Methods

This section presents the method in three connected layers. We first construct a source-specific circuit-level noise model in which idling noise, single-qubit control noise, photon-induced dephasing, and flux-induced phase noise are retained as distinct components within a unified description. We then embed sampled flux-induced phase offsets into the circuit-execution timeline by partitioning the timeline into segments parameterized by the temporal scale $\tau_{c}$ . Finally, we organize these ingredients into an execution-level evaluation workflow that produces ideal and noisy outputs under otherwise matched circuit and noise settings, so that the effect of temporal correlation can be examined through controlled comparison.

### 2.1. Circuit-Level Noise Modeling Framework

The noise model in this work is organized as a set of source-specific circuit-level components rather than as a single effective channel. Idling noise, single-qubit control noise, photon-induced dephasing, and flux-induced phase noise enter the circuit execution through different mechanisms and are therefore retained as distinct components in a unified circuit-level framework. This separation makes the role of the segmented flux-induced phase component explicit while preserving the fixed composite-noise background used in the numerical experiments.

The source-specific components are chosen to reflect distinct error mechanisms reported for superconducting transmon circuits and adopted here in circuit-level form. In the reference implementation of O’Brien et al. [3], these noise channels are expressed in Pauli transfer matrix (PTM) form; in the present work, we use abstract channel and operation notation in the exposition, while the corresponding simulator-level actions are implemented through Kraus-form channels. Idling noise is used to represent relaxation and background pure dephasing accumulated during waiting periods and during the effective durations assigned to gates. Consistent with the reference treatment described above, we represent idling noise using an amplitude–phase damping channel.

(1) $$E_{\mathrm{AP}}\left(t\right)=E_{T_{1}}\left(t\right)\circ E_{T_{\phi }}\left(t\right),\;p_{1}=1-e^{-t/T_{1}},\;p_{\phi }=1-e^{-t/T_{\phi }},$$

where *t* denotes the relevant execution interval; $T_{1}$ and $T_{\phi }$ are the relaxation and pure-dephasing times, respectively; $e^{-t/T_{1}}$ and $e^{-t/T_{\phi }}$ are the corresponding survival factors; and $p_{1}$ and $p_{\phi }$ quantify the corresponding relaxation and pure-dephasing accumulated over the interval *t*. Photon-induced dephasing is introduced to capture the residual effect of readout-resonator photons. At the circuit level, its contribution is determined by the relevant post-measurement interval through the dephasing factor

(2) $$p_{\phi ,\mathrm{photon}}\left(t_{1},t_{2}\right)=\exp\;\left(-\int_{t_{1}}^{t_{2}}\Gamma_{d}\left(t\right)\;dt\right),$$

where $\left[t_{1},t_{2}\right]$ is the post-measurement interval associated with the operation under consideration and $\Gamma_{d}\left(t\right)$ is the corresponding photon-induced dephasing rate. In the circuit-level tests below, this photon-induced dephasing channel is used as a prescribed time-dependent background component of the composite noise setting. It should not be interpreted as implying that the CPMG or RB test circuits themselves contain intermediate measurements that generate new residual photons. Instead, the channel supplies a fixed background dephasing contribution whose parameters are kept unchanged when the segmented flux-induced phase component is varied. For the driven $R_{y}\left(\pi /2\right)$ rotations considered here, we adopt a gate-specific noise model rather than absorb all imperfections into generic depolarization; specifically, the ideal driven rotation is combined with amplitude-phase damping and an anisotropic depolarizing correction

(3) $$E_{R_{y}}=E_{\mathrm{AP}}\left(t_{1}/2\right)\circ U_{y}\left(\pi /2\right)\circ E_{\mathrm{dep}}\circ E_{\mathrm{AP}}\left(t_{1}/2\right),$$

where $t_{1}$ is the effective duration of the driven single-qubit gate, $U_{y}\left(\pi /2\right)$ denotes the ideal π/2 rotation about the *y*-axis, and $E_{\mathrm{dep}}$ is parameterized by $p_{\mathrm{axis}}$ and $p_{\mathrm{plane}}$ , which quantify an anisotropic depolarizing correction: in the Bloch-sphere picture, the gate action is contracted by a factor $1-p_{\mathrm{axis}}$ along the driven rotation axis and by a factor $1-p_{\mathrm{plane}}$ in the transverse plane. Flux-induced phase noise is treated separately. When a transmon is moved away from its sweet spot by flux pulsing, it becomes first-order-sensitive to flux fluctuations and therefore acquires an additional phase shift

(4) $$\delta \phi_{q}=-2\pi \;\tau_{\mathrm{flux}}\frac{\partial f_{q}}{\partial \Phi }\;\delta \Phi ,$$

where $\tau_{\mathrm{flux}}$ is the effective duration of the relevant flux-sensitive interval, $f_{q}$ is the qubit frequency, Φ is the applied flux bias, and δΦ is the corresponding flux deviation. In the reference treatment, this effect is approximated as quasi-static within a single run and varying between runs; the same physical mechanism may also contribute to phase errors in multiqubit flux-pulsed operations, but the present numerical evaluation focuses on single-qubit circuits. At the circuit level, however, the present work does not track a continuous time-dependent flux perturbation directly. Instead, its net effect is represented as additional phase angles applied to flux-sensitive gate operations. Accordingly, throughout the remainder of this work, *flux-induced phase noise* refers to the circuit-level phase-offset contribution used to represent the effect of underlying flux fluctuations on flux-sensitive operations. It does not denote a complete continuous-time or device-level model of all flux-noise mechanisms. In this representation, the case in which a single sampled phase angle is held fixed throughout an entire circuit run is recovered as a special case, while the construction introduced below extends this description to a piecewise-constant collection of phase angles distributed along the execution timeline.

Once these source-specific components are defined, they are incorporated into a unified circuit-level model. Once an explicit timing description of the circuit is available, idling noise and driven single-qubit control noise can be assigned according to operation duration, while photon-induced dephasing is determined by the relevant post-measurement interval. Flux-induced phase noise requires an additional assignment step beyond the timing-based treatment of the other components. The execution timeline is partitioned into segments of duration $\tau_{c}$ , and sampled phase-angle offsets are assigned to the corresponding flux-sensitive gate operations. Here, $\tau_{c}$ denotes the segment duration and serves as the operational temporal-memory parameter of the construction. Varying $\tau_{c}$ changes the temporal organization of the sampled phase offsets while the remaining circuit and noise settings are kept fixed, allowing the resulting circuit-level changes to be attributed to the imposed memory structure. The specific timing representation and segment-assignment rule are introduced in Section 2.2.

### 2.2. Segmented Correlation-Time Embedding of Flux-Induced Phase Noise

The flux-noise treatment adopted in this work is inspired by the quasi-static approximation used by O’Brien et al. [3]. In that description, the net phase effect of flux noise is treated as effectively constant during a single circuit run and is allowed to vary only between repeated runs. This approximation reflects the long-correlation character of low-frequency flux noise and provides a circuit-level description of the resulting phase perturbation on flux-sensitive operations. In the present work, we generalize this idea to a piecewise-constant, segment-based flux-noise construction in which the sampled phase angle is represented over finite time windows rather than fixed over the whole run. Under this interpretation, the single-run constant treatment is recovered as a special case of the present model. This segmentation is introduced at the level of circuit timing, so that phase-angle offsets are assigned according to the execution timeline of the circuit.

**Figure 1:**
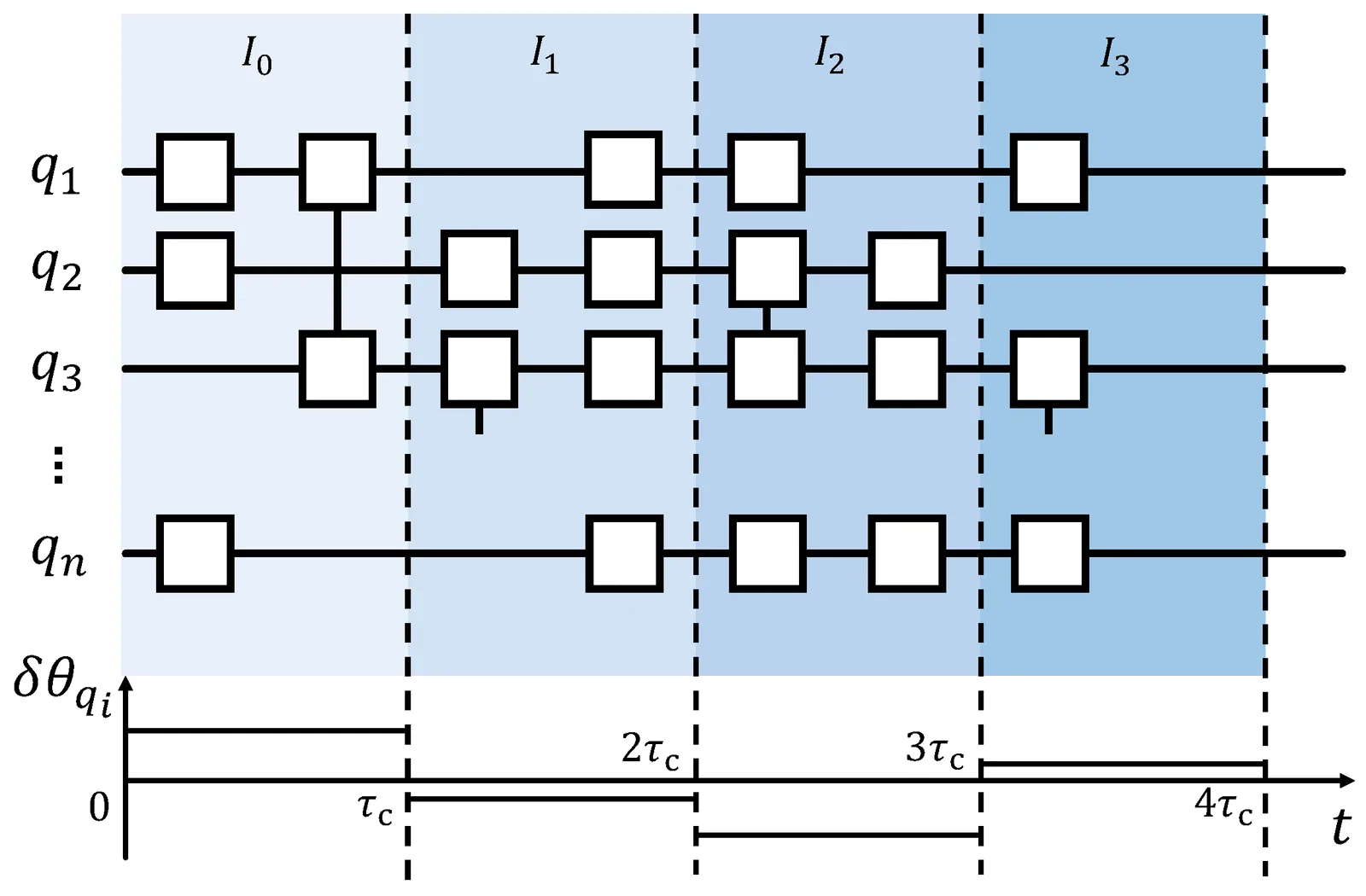
Schematic illustration of the segmented correlation-time construction for flux-induced phase noise. The circuit-execution timeline is partitioned into contiguous segments $I_{0},I_{1},\ldots$ of width $\tau_{c}$ . For each segment and each qubit, one sampled phase-angle offset is drawn and kept constant within that segment. Each flux-sensitive physical gate operation is associated with the segment determined by its execution interval and receives the sampled offset assigned to the corresponding qubit and segment according to the gate-level rule of the model. The lower panel shows a representative stepwise-constant sampled profile for a single qubit; analogous profiles are sampled independently for the other qubits.

To implement this construction, the circuit is first embedded into an explicit timing representation. Each operation is associated with an execution start time and an effective duration. Explicit waiting intervals preserve their physical durations; virtual-*Z* updates, implemented as zero-duration frame changes, are treated as zero-duration operations; and the remaining single- and two-qubit gates are assigned fixed effective durations according to the simulation setting. Once this timing description is available, the full execution timeline is partitioned into contiguous segments of width $\tau_{c}$ , where $\tau_{c}$ denotes the segment duration and serves as the operational temporal-memory parameter of the construction. For each segment and each qubit, one phase-angle offset is sampled and held constant throughout that segment. In the present implementation, these segment variables are sampled independently for distinct segment–qubit pairs. Each gate operation is then mapped to the segment associated with its execution interval, and the sampled offset corresponding to that segment and qubit is applied according to the gate-level rule of the model. Figure 1 summarizes this segment-based temporal assignment.

To make this construction explicit, let the circuit-execution timeline be partitioned into segments

(5) $$I_{s}=\left[s\tau_{c},\left(s+1\right)\tau_{c}\right),\;s=0,1,\ldots ,S-1,$$

where $\tau_{c}$ is the segment duration and *S* is the number of segments required to cover the total execution interval. For each flux-sensitive gate $g_{k}$ , let $t_{k}$ and $d_{k}$ denote its execution start time and effective duration, respectively. We assign $g_{k}$ to a segment index

(6) $$s\left(k\right)=\arg\max_{s}\left|\left[t_{k},t_{k}+d_{k}\right)\cap I_{s}\right|,$$

that is, to the segment with the largest temporal overlap with the gate interval. This maximum-overlap convention is used as the default deterministic assignment rule in the main simulations. For operations whose execution interval lies entirely within a single segment, all standard assignment conventions coincide. The assignment convention can therefore affect only operations whose execution interval crosses a segment boundary. The use of the largest-overlap segment assigns such an operation to the segment that covers the largest fraction of its execution interval, while preserving a single segment label for the operation-level phase insertion rule. For each sampled realization, we sample one Gaussian phase angle for each segment and qubit,

(7) $$\delta \theta_{s,q}∼N\left(0,\sigma^{2}\right),\;s=0,1,\ldots ,S-1,$$

where *q* labels the qubit and σ is the standard deviation of the sampled segment-wise phase angle. The samples have zero mean and are independent for distinct segment–qubit pairs.

At the gate level, the sampled segment variable is applied following the gate-execution phase-error picture for flux-sensitive operations in flux-tunable transmon circuits. In this picture, low-frequency flux fluctuations during gate execution can be represented by a quasi-static flux offset, and the flux dependence of the qubit transition frequency converts this offset into an accumulated phase perturbation over the gate interval. The sampled variable $\delta \theta_{s,q}$ is therefore used as the effective flux-induced phase accumulated by qubit *q* in segment *s*.

For a single-qubit flux-sensitive operation $U_{g}$ acting on qubit *q* and assigned to segment *s*, the corresponding noisy operation is written as

(8) $${\tilde{U}}_{g,s,q}=R_{z}\left(\delta \theta_{s,q}\right)U_{g},\;R_{z}\left(\delta \theta_{s,q}\right)=\exp\;\left(-\frac{i}{2}\delta \theta_{s,q}Z\right).$$

Equivalently, at the density-matrix level,

(9) $$\rho \mapsto R_{z}\left(\delta \theta_{s,q}\right)U_{g}\rho U_{g}^{†}R_{z}^{†}\left(\delta \theta_{s,q}\right).$$

This form expresses the net flux-induced phase error accumulated during the gate execution as an additional *Z*-axis phase rotation.

Operations that do not correspond to a physical gate-execution interval are excluded from this rule. In particular, zero-duration virtual frame updates, including virtual-*Z* operations, are not assigned an additional sampled flux-induced phase. Explicit waiting intervals are handled by the idle-noise channel rather than by this gate-level phase insertion rule. Since the numerical experiments in this work evaluate single-qubit CPMG and RB circuits, we do not include a separate two-qubit local or conditional phase-insertion rule in the main model; extension to entangling operations is left for future work.

To characterize the temporal statistics associated with this construction, we define the corresponding piecewise-constant process as

(10) $$C_{\theta ,qq^{\prime }}^{\mathrm{seg}}\left(t,t^{\prime }\right)=\langle \delta \theta_{q}\left(t\right)\delta \theta_{q^{\prime }}\left(t^{\prime }\right)\rangle =\sigma^{2}\delta_{qq^{\prime }}\delta_{s\left(t\right),s\left(t^{\prime }\right)}.$$

where 〈·〉 denotes the ensemble average over the sampled segment variables and $\delta_{qq^{\prime }}$ is the Kronecker delta. Because the segment-wise phase variables are zero mean, Equation (10) is both their second-moment function and their covariance. After a physical operation *g* is assigned to the segment s(g) , the same covariance rule induces the operation-level form introduced below. For the same qubit, the covariance is $\sigma^{2}$ when *t* and $t^{\prime }$ fall in the same segment and zero otherwise; different qubits are independent at the level of the segment variables. Since the segment grid is anchored to the beginning of each circuit execution, $C_{\theta ,qq^{\prime }}^{\mathrm{seg}}\left(t,t^{\prime }\right)$ is an execution-referenced two-time covariance and is not assumed to depend only on $t-t^{\prime }$ .

Equation (10) makes the blockwise memory structure explicit: for each qubit, the effective phase offset is shared within a segment and refreshed discretely at the segment boundary. This refresh is a modeling operation applied to the circuit-level phase variable, rather than a claim that the underlying physical flux waveform undergoes an instantaneous jump. The relation of $\tau_{c}$ to gate durations, pulse spacings, and the total circuit execution time governs how scheduled operations sample this blockwise temporal structure and, for frequency-selective pulse sequences, provides the basis for interpreting the resulting circuit response. In this way, the construction isolates the execution-level consequences of varying temporal memory under controlled conditions. Consequently, we do not assign a unique stationary power spectral density to this fixed-origin segmented process. Defining such a spectrum would require an additional stationarization convention, such as averaging over a random segment origin, which is not the process simulated in the present work.

Through the linearized flux-to-phase relation in Equation (4), the phase-fluctuation scale can be related to an underlying flux-fluctuation scale. For a given qubit at a fixed operating point and flux-sensitive duration, the corresponding standard deviations satisfy

(11) $$\sigma_{\phi }=2\pi \tau_{\mathrm{flux}}\left|\frac{\partial f_{q}}{\partial \Phi }\right|\sigma_{\Phi },$$

where $\sigma_{\Phi }$ and $\sigma_{\phi }$ denote the standard deviations of the flux deviation and the resulting phase shift, respectively.

To relate the segment-wise phase parameterization to a continuous-time physical noise description, let $\delta \Phi_{q}\left(t\right)$ denote a zero-mean continuous-time flux fluctuation, and consider a flux-sensitive physical operation *g* acting on qubit $q_{g}$ over the execution interval $I_{g}$ . Near a fixed operating point, a small flux fluctuation produces the frequency fluctuation

(12) $$\delta \omega_{q}\left(t\right)=2\pi \frac{\partial f_{q}}{\partial \Phi }\delta \Phi_{q}\left(t\right).$$

For the longitudinal phase-noise channel considered here, the phase accumulated by operation *g* can be written as

(13) $$\vartheta_{g}=\int_{I_{g}}y_{g}\left(t\right)\delta \omega_{q_{g}}\left(t\right)\;dt,$$

where $y_{g}\left(t\right)$ denotes the effective time-dependent sensitivity of operation *g* over $I_{g}$ . The covariance between the accumulated phases of two operations is then

(14) $$C_{gh}^{\mathrm{phys}}\equiv \mathrm{Cov}\left(\vartheta_{g},\vartheta_{h}\right)=\int_{I_{g}}dt\int_{I_{h}}dt^{\prime }\;y_{g}\left(t\right)y_{h}\left(t^{\prime }\right)C_{\omega ,q_{g}q_{h}}\left(t,t^{\prime }\right),$$

where

(15) $$C_{\omega ,qq^{\prime }}\left(t,t^{\prime }\right)=E\;\left[\delta \omega_{q}\left(t\right)\delta \omega_{q^{\prime }}\left(t^{\prime }\right)\right].$$

Equation (14) applies without requiring stationarity of the underlying continuous-time process.

If the underlying frequency fluctuation is additionally assumed to be stationary, so that $C_{\omega ,qq^{\prime }}\left(t,t^{\prime }\right)=C_{\omega ,qq^{\prime }}\left(t-t^{\prime }\right)$ , we define the corresponding two-sided cross-spectral density as

(16) $$S_{\omega ,qq^{\prime }}\left(\omega \right)=\int_{-\infty }^{\infty }C_{\omega ,qq^{\prime }}\left(\Delta t\right)e^{i\omega \Delta t}\;d\Delta t.$$

With this convention, the same gate-level covariance can be expressed as

(17) $$C_{gh}^{\mathrm{phys}}=\frac{1}{2\pi }\int_{-\infty }^{\infty }S_{\omega ,q_{g}q_{h}}\left(\omega \right)Y_{g}^{*}\left(\omega \right)Y_{h}\left(\omega \right)\;d\omega ,\;Y_{g}\left(\omega \right)=\int_{I_{g}}y_{g}\left(t\right)e^{i\omega t}\;dt.$$

For g=h , this expression gives the accumulated phase variance of operation *g*. This is the standard filter-function connection between a stationary noise spectrum, the temporal structure of the applied control, and the resulting phase fluctuations [9,17,18].

The present segmented construction acts directly at the operation level. Rather than first generating a continuous-time frequency-noise trajectory, it assigns the effective accumulated phase

(18) $$\vartheta_{g}^{\mathrm{seg}}=\delta \theta_{s\left(g\right),q_{g}}$$

to each eligible physical operation *g*. Its operation-level phase covariance is therefore

(19) $$\begin{array}{cc} C_{gh}^{\mathrm{seg}} & \equiv \mathrm{Cov}\;\left(\vartheta_{g}^{\mathrm{seg}},\vartheta_{h}^{\mathrm{seg}}\right)\\ \; & =E\;\left[\delta \theta_{s\left(g\right),q_{g}}\delta \theta_{s\left(h\right),q_{h}}\right]\\ \; & =\sigma^{2}\delta_{q_{g}q_{h}}\delta_{s(g),s(h)}. \end{array}$$

Equation (19) is the operation-level counterpart of Equation (10): the first Kronecker delta enforces the independent-qubit sampling rule, while the second enforces sharing of the same phase variable only between operations assigned to the same segment.

Within this execution-level parameterization, σ sets the standard deviation of each segment-wise phase variable. Throughout a $\tau_{c}$ sweep, every segment variable retains the distribution $\delta \theta_{s,q}∼N\left(0,\sigma^{2}\right)$ , while $\tau_{c}$ changes the segment map s(g) and hence the block structure of $C_{gh}^{\mathrm{seg}}$ . The comparison therefore isolates the circuit-level effect of varying the temporal range over which operations share a phase sample drawn from the same fixed distribution. A device-specific spectral correspondence can subsequently be established by specifying the continuous-time noise model, the control sensitivity, and the calibration criterion used to match the physical covariance $C_{gh}^{\mathrm{phys}}$ to its segmented approximation $C_{gh}^{\mathrm{seg}}$ .

When $\tau_{c}$ is comparable to or longer than the total execution time of a circuit run, each relevant qubit effectively carries one sampled phase angle throughout that run, recovering the whole-run quasi-static limit used in the reference treatment. As $\tau_{c}$ decreases, the execution timeline contains progressively more segments, and different portions of the same circuit can therefore experience different sampled phase offsets. When $\tau_{c}$ is short relative to the resolved circuit time scales, the effective phase offsets may be viewed as exhibiting white-noise-like behavior in the limited sense of rapid segment-to-segment variation and short operational memory. The segmented embedding enables the same logical circuit and the same non-flux noise setting to be evaluated under different segment-duration settings without changing the remaining simulation conditions.

The construction therefore combines physics-motivated ingredients with explicit modeling choices made for controlled circuit-level comparison. The linearized flux-to-phase relation, the quasi-static limiting case, and the use of the circuit-execution timeline provide the physical basis of the model. Fixed-width segmentation, independent Gaussian refreshes across segments, and qubit-wise independent segment variables are controllable abstractions that allow the temporal-memory parameter to be varied independently of the remaining circuit and noise settings.

The present construction and standard continuous-time noise models can now be compared at the level of the induced gate-phase covariance. A band-limited 1/f -type model begins from a stationary spectrum of the form

(20) $$S_{\omega }^{1/f}\left(\omega \right)=\frac{A_{\omega }}{{\left|\omega \right|}^{\alpha }},\;\omega_{l}\leq \left|\omega \right|\leq \omega_{h},$$

with explicitly chosen low- and high-frequency cutoffs [8]. Together with the control sensitivities $Y_{g}\left(\omega \right)$ , this spectrum determines $C_{gh}^{\mathrm{phys}}$ through Equation (17). Such a model contains a broad distribution of temporal scales rather than a single correlation time.

An Ornstein–Uhlenbeck (OU) frequency-noise process instead has the stationary exponential covariance

(21) $$C_{\omega }^{\mathrm{OU}}\left(\Delta t\right)=\nu_{\omega }^{2}\exp\;\left(-\frac{\left|\Delta t\right|}{\tau_{\mathrm{OU}}}\right),$$

and the corresponding Lorentzian spectrum

(22) $$S_{\omega }^{\mathrm{OU}}\left(\omega \right)=\frac{2\nu_{\omega }^{2}\tau_{\mathrm{OU}}}{1+\omega^{2}\tau_{\mathrm{OU}}^{2}}$$

under the two-sided spectral convention used above [19]. In this case, $\tau_{\mathrm{OU}}$ is defined by the exponential decay of a stationary continuous-time covariance.

The segmented construction specifies a different covariance structure directly at the execution level: $C_{gh}^{\mathrm{seg}}$ is blockwise constant, with operations assigned to the same segment on the same qubit sharing one phase variable. Its parameter $\tau_{c}$ sets the width of these execution-aligned covariance blocks. Consequently, the present segmented construction, an OU model, and a band-limited 1/f -type model encode temporal structure in different mathematical forms and become quantitatively comparable only after a specific covariance- or response-matching criterion is chosen.

Experimentally reconstructed spectra or time-domain noise records provide a device-specific route to the physical covariance $C_{gh}^{\mathrm{phys}}$ . Once the operating-point sensitivity and the relevant control functions are specified, an effective pair $\left(\sigma ,\tau_{c}\right)$ can be selected by matching quantities such as the accumulated phase variance, a chosen set of gate-phase covariances, or a measured circuit response. Noise spectroscopy based on dynamical decoupling provides one experimental route for obtaining the spectral information required for such a calibration [9]. The selected effective parameters may depend on the matching observable and time window because the segmented construction compresses a potentially multiscale physical covariance into a two-parameter execution-level representation.

### 2.3. Execution-Level Evaluation Workflow

With the source-specific circuit-level noise components and the segmented correlation-time construction for flux-induced phase noise defined, the remaining task is to integrate them into a controlled execution-level evaluation workflow. Starting from a circuit specification and a chosen parameter setting, the workflow produces both an ideal reference output and the corresponding noisy circuit output under the same logical circuit definition. In this way, the evaluation is not based on isolated noisy realizations alone, but on a consistent comparison between the ideal and noisy behavior of the same circuit specification.

For a given circuit and parameter setting, the workflow first instantiates the logical circuit and, when required, applies the compilation procedure specified for the target circuit family. The ideal reference is then generated from the same circuit specification without noise, while the noisy execution is obtained by attaching the composite circuit-level noise model introduced in Section 2.1. For the non-flux components, the noise contribution is determined once the circuit timing, operation types, and relevant timing intervals are fixed. For the flux-noise component, the construction introduced in Section 2.2 assigns segment-wise sampled phase angles according to the chosen value of $\tau_{c}$ , and these phase angles are applied to the corresponding flux-sensitive gate operations according to the gate-level noise rule of the model. The resulting noisy circuit output is then evaluated against the ideal reference through the metrics defined later in this paper.

A central design principle of this workflow is controlled comparison. When $\tau_{c}$ is varied, the logical circuit, the non-flux noise setting, the gate-level rule that applies the sampled phase angles to flux-sensitive operations, and the evaluation metrics are all kept fixed. What changes is only the temporal organization of the segmented flux-induced phase process along the execution timeline. This ensures that differences observed in the circuit outputs can be attributed to the change in temporal structure, rather than to unrelated changes in circuit definition or overall noise configuration.

The workflow further separates simulation execution from statistical post-processing. For each parameter setting and each sampled realization, the execution stage produces raw circuit-level outputs together with the metadata needed for later aggregation. Statistical summarization, block averaging, error-bar estimation, and figure generation are then carried out in a separate post-processing stage without rerunning the underlying noisy-circuit simulations. This separation is methodologically useful for the present study because it allows the same set of raw realizations to support multiple summary strategies and consistency checks while preserving a clear distinction between simulated data generation and later statistical interpretation.

## 3. Experimental Setup

### 3.1. Composite Noise Setting

All experiments in this work are performed under the composite circuit-level noise model introduced in Section 2 rather than under an isolated flux-noise-only setting. The simulated noise background includes idling noise, single-qubit control noise, photon-induced dephasing, and the present flux-induced phase-noise component within a unified framework. Here, “composite” denotes the simultaneous inclusion of these source-specific components under a common circuit-timing representation, with each component retaining its own operation- or interval-level application rule. This choice places the flux-noise component in the presence of other circuit-relevant error channels and avoids interpreting its effect in an otherwise idealized noise environment.

Within this composite model, the flux-noise component is the only noise source whose temporal correlation scale is scanned systematically in the main experiments. The remaining noise channels serve as a common background setting unless otherwise specified. In selected experiments, the fluctuation strength σ is additionally varied in order to examine how the dependence on $\tau_{c}$ changes with the magnitude of the sampled phase-angle perturbation. The common timing and noise parameters used in the evaluation are summarized in Table 1.

Accordingly, the main experiments are designed to isolate the effect of the temporal organization of the flux-induced phase component. The remaining noise channels are retained as fixed execution-relevant backgrounds and are not independently scanned to establish cross-channel interaction effects in the present study. In particular, the photon-induced dephasing parameters listed in Table 1 are held fixed across the $\tau_{c}$ sweeps. This channel therefore contributes to the absolute fidelity or survival-probability baseline, but it does not introduce an independent $\tau_{c}$ -dependent control parameter.

### 3.2. Circuits and Correlation-Time Settings

The evaluation is carried out on two classes of single-qubit circuits with different structural roles. The first is a single-qubit Carr–Purcell–Meiboom–Gill (CPMG) sequence. The circuit applies an initial Hadamard gate, a half waiting interval, 100 Pauli-*X* refocusing pulses separated by waiting intervals of 400 ns, a final half interval, and a Hadamard gate for readout. The refocusing pulses reverse the sign of phase accumulation induced by slowly varying detuning, while their spacing provides a defined circuit time scale against which the segmented phase process is sampled. CPMG therefore provides a structured probe of the relation between pulse timing and the temporal-memory parameter $\tau_{c}$ . The circuit is used directly without an additional compilation stage, and its output is evaluated using the circuit-output fidelity defined in Section 3.3. For this directly constructed CPMG circuit, the sampled flux-induced phase offsets are applied to the physical *H* and *X* gate executions according to the gate-level rule in Section 2.2, while the explicit waiting intervals are described by the idle-noise channel and are not assigned an additional gate-level flux-induced phase. The second class consists of standard single-qubit randomized benchmarking (RB) circuits [20]. For each nominal depth *m*, an RB circuit contains *m* gates randomly sampled from the 24-element single-qubit Clifford group, followed by a recovery Clifford that ideally returns the qubit to its initial state. To express these abstract Clifford gates in native operations to which the circuit-level noise channels can be applied while avoiding compiler-induced cancellation or merging across successive Cliffords, each Clifford is subsequently treated as an independent compilation block. CPMG and RB therefore probe the segmented phase process from complementary perspectives: CPMG uses a regular refocusing pattern with prescribed pulse spacing to resolve the interaction between temporal memory and a controlled circuit time scale, whereas RB uses randomized gate sequences of increasing depth to examine how the same temporal structure is accumulated and expressed in a more generic circuit setting.

For the CPMG experiments, the present flux-noise model is evaluated over the correlation-time grid

(23) $$\tau_{c}\in \left\{20,40,60,80,120,200,400,800,1200,2400,5000,8000,20,000,40,000\right\}\;\mathrm{ns},$$

and three fluctuation strengths are considered,

(24) σ∈{0.03,0.06,0.10}.

These values are used as controlled circuit-level phase-fluctuation settings for evaluating amplitude sensitivity; they are not presented as a calibration to one specific superconducting device. For each fixed value of σ , the $\tau_{c}$ sweep retains the same segment-level phase variance and changes only the temporal organization of the sampled phase offsets.

To check that the observed $\tau_{c}$ -dependent behavior is not an artifact of the deterministic segment boundary or of the maximum-overlap assignment rule, we also perform a boundary-assignment robustness check on the representative CPMG setting. In this check, the default fixed-origin, maximum-overlap construction is compared with variants using a random segment origin and alternative assignment conventions, including start time, midpoint, and overlap-weighted random assignment. The same logical circuit, noise parameters, and sample size are otherwise kept unchanged.

For each $\left(\tau_{c},\sigma \right)$ setting in the CPMG experiments, the reported quantity is estimated from 2000 independently sampled noisy realizations. The corresponding output metric is the circuit-output fidelity under the fixed composite-noise background described in Section 3.1. The CPMG and RB test circuits considered here do not contain intermediate measurement operations. The photon-induced dephasing term in the composite model is therefore not generated by measurements inside these test circuits; it is included as part of the fixed background environment described in Section 3.1.

For the randomized benchmarking experiments, the same $\tau_{c}$ grid is used so that the role of the flux-noise time scale can be compared across circuit families on a common axis. In the fixed-depth survival study, the fluctuation strength is set to σ=0.10 , and the RB depths are

(25) m∈{64,256,1024}.

In the decay-shape study, the RB depth list is extended to

(26) m∈{2,4,8,12,16,24,32,48,64,96,128,192,256,384,512,768,1024}.

For each $\left(\tau_{c},m\right)$ setting in the RB experiments, the reported survival quantity is estimated from 300 independently sampled sequence–noise pairs. Each pair consists of an independently generated RB sequence and an independently sampled noisy realization of the segmented flux-induced phase process under the same fixed background noise parameters. The resulting mean therefore estimates the joint ensemble average over random RB sequences and noise realizations.

For the standard single-qubit RB experiments, the abstract Clifford sequence is compiled to the target native gate set using a weak blockwise compilation procedure. In this procedure, each moment of the original RB circuit is treated as an independent Clifford block and is decomposed into native single-qubit *Z*- and *Y*-axis rotations without cross-block merging, large-scale timing reshaping, operation reordering, or as-soon-as-possible (ASAP)-style rescheduling. The purpose of this choice is to preserve the sequence-length structure of RB while still expressing the circuit in a gate set to which the operation-specific noise models can be attached. Gate-dependent effects are retained at the compiled-operation level through the operation-specific durations and noise channels of the composite model, rather than being replaced by a single gate-independent error channel. Under the adopted *Z*–*Y*–*Z* decomposition, each Clifford block contains at most one duration-bearing *Y* rotation, while the accompanying *Z* rotations are implemented as zero-duration virtual frame updates. Accordingly, the sampled flux-induced phase offset is applied to the physical *Y* rotation in each compiled Clifford block when such a rotation is present. The surrounding *Z* rotations are zero-duration virtual frame updates and are therefore excluded from the gate-level flux-induced phase rule. For a sequence of nominal depth *m*, the compiled circuit therefore contains at most m+1 duration-bearing rotations, including the recovery Clifford. Its exact execution duration remains sequence-dependent, because some Clifford blocks require no nontrivial *Y* rotation, but both its upper bound and its ensemble-average duration scale linearly with *m*. The execution timeline used by the segmented noise model is constructed from this compiled circuit. As a result, the nominal RB depth remains meaningfully associated with the executed circuit structure, which is important for comparisons of depth-dependent responses to the correlation-time parameter $\tau_{c}$ .

### 3.3. Metrics and Comparison Protocol

Different output metrics are used for different circuit families according to the role of each experiment. For the CPMG experiments, we report the standard-state fidelity between the noisy output state and the ideal output state,

(27) $$F_{\mathrm{state}}\left(\rho_{\mathrm{out}},\rho_{\mathrm{ideal}}\right)={\left(\mathrm{Tr}\sqrt{\sqrt{\rho_{\mathrm{ideal}}}\;\rho_{\mathrm{out}}\;\sqrt{\rho_{\mathrm{ideal}}}}\right)}^{2}.$$

Here $\rho_{\mathrm{out}}$ is the simulated final density matrix and $\rho_{\mathrm{ideal}}$ is the ideal final density matrix of the same circuit. In the CPMG experiments considered here, the ideal output is a pure target state. Therefore, the above expression reduces to the target-state overlap

(28) $$F_{\mathrm{state}}=\mathrm{Tr}\left(\rho_{\mathrm{ideal}}\rho_{\mathrm{out}}\right)=\langle \psi_{\mathrm{ideal}}|\rho_{\mathrm{out}}|\psi_{\mathrm{ideal}}\rangle .$$

For a |0〉 target, this is also the return probability $\langle 0|\rho_{\mathrm{out}}|0\rangle$ . For each CPMG setting, the reported output-state fidelity is the sample mean over 2000 independently sampled noisy realizations, and the error bars indicate the standard error of the mean (SEM). For two independently sampled means, we estimate the standard error of their difference as $\sqrt{{\mathrm{SEM}}_{a}^{2}+{\mathrm{SEM}}_{b}^{2}}$ . The endpoint comparisons in Section 4.1 use this expression with a=20ns and b=40,000ns . For an individual RB sequence, the survival probability is defined as the population of the initial state after application of the recovery Clifford, $P_{\mathrm{surv}}=\langle \psi_{0}|\rho_{\mathrm{out}}|\psi_{0}\rangle$ , where $|\psi_{0}\rangle$ is the prepared initial state and $\rho_{\mathrm{out}}$ is the final noisy output state. In the present implementation, $|\psi_{0}\rangle =|0\rangle$ , so that $P_{\mathrm{surv}}=\langle 0|\rho_{\mathrm{out}}|0\rangle$ . At each $\left(\tau_{c},m\right)$ setting, the reported mean survival probability is the sample mean of $P_{\mathrm{surv}}$ over 300 independently sampled sequence–noise pairs. The corresponding error bar is the standard error of this sample mean. This uncertainty therefore reflects the finite-sample variation in the joint sequence–noise ensemble, rather than a separate decomposition into sequence-only and noise-only variance components. When comparing adjacent $\tau_{c}$ points in the fixed-depth RB sweeps, we assess the size of a local change relative to the same independent-mean-difference standard error. This comparison is used only to distinguish visually small local fluctuations from the main depth-dependent trend. In the RB decay analysis, the same survival data are further summarized through a single-exponential reference fit of the form

(29) $$P\left(m\right)=Ap^{m}+B,$$

where *m* denotes the RB sequence depth, *p* is the fitted decay parameter, and *A* and *B* are fit coefficients. In addition, the root-mean-square error and the maximum absolute residual are extracted from the fit residuals for later analysis. To quantify the finite-sample uncertainty of the fitted RB summaries, we use a nonparametric bootstrap over the same sequence–noise pairs. For each bootstrap replicate, the 300 sequence–noise values at each depth *m* are resampled with replacement, the mean survival curve is recomputed, and the single-exponential reference fit is repeated. The bootstrap distribution is then used to estimate standard errors and percentile confidence intervals for the fitted decay parameter, EPC, average fidelity, RMSE, and maximum absolute residual.

In addition, to distinguish the two sources of finite-sample variation, we perform a representative nested diagnostic on selected RB settings. For each selected $\left(\tau_{c},m\right)$ , we generate 50 independent RB sequences and simulate 20 independent noise realizations for each fixed sequence. This produces survival values $P_{ij}=P\left(S_{i},\xi_{ij}\right)$ , from which we estimate the sequence-to-sequence component through the variance in the sequence-averaged survivals and the noise-realization component through the average within-sequence variance. This diagnostic is used to characterize the relative contributions of the two sources of variation on representative settings; the main RB curves remain based on the 300 sequence–noise pairs described above.

For the statistical presentation of the RB data, each plotted point represents the sample mean over the corresponding ensemble of sequence–noise pairs, and the error bars indicate the standard error of the mean. All comparisons in the evaluation are organized according to a controlled-comparison protocol. Within a given $\tau_{c}$ sweep, the logical circuit, the non-flux noise setting, the gate-level rule that applies sampled phase-angle offsets to gate operations, and the metric definition are kept fixed. What varies is only the flux-induced phase-noise parameter under study, namely $\tau_{c}$ and, in selected CPMG experiments, the fluctuation strength σ .

The simulation scripts, configuration files, random seeds, post-processing procedures, and raw numerical outputs used for the revised CPMG and RB analyses are provided through the public repository cited in the Data Availability Statement.

## 4. Evaluation

We organize the evaluation in three successive steps. We first study a structured single-qubit CPMG sequence and examine how its output fidelity depends on the correlation-time parameter $\tau_{c}$ and the fluctuation strength σ . Because the CPMG pulse pattern has a frequency-selective character, this experiment reveals how the segmented flux-induced phase process is sampled by a refocusing circuit and how the resulting response changes from the short-correlation regime to the high- $\tau_{c}$ plateau.

We then turn to standard single-qubit randomized benchmarking. At a fixed circuit depth, we examine how the mean survival probability changes with $\tau_{c}$ and show that the onset of the high- $\tau_{c}$ plateau shifts systematically with circuit depth. We next analyze the full RB decay curves at representative correlation times and show that increasing $\tau_{c}$ progressively reshapes the decay profile, producing a more slowly decaying tail at intermediate and large depths. Finally, we quantify this deviation from the standard single-exponential RB picture through the fitted decay parameter *p* together with residual-based diagnostics. Taken together, these results show that the impact of the segmented flux-induced phase process is governed jointly by the correlation-time parameter $\tau_{c}$ , the temporal structure of the circuit, and the way in which the circuit samples the noise.

### 4.1. CPMG Fidelity Under Different Flux-Induced Phase-Noise Strengths

We first examine a single-qubit CPMG sequence in order to study how the response to the modeled flux-induced phase noise changes with both the model correlation-time parameter $\tau_{c}$ and the fluctuation strength σ . In the present experiments, the circuit contains 100 refocusing pulses with a waiting duration of 400 ns between pulses, and the fidelity metric defined in Section 3.3 is evaluated under the fixed composite-noise background. Because CPMG is a structured refocusing sequence, its pulse pattern does not simply accumulate phase noise passively; rather, it gives the circuit a frequency-selective character. In filter-function terms, the pulse spacing makes the sequence most sensitive to noise components around characteristic frequencies set by the pulse pattern, together with higher-harmonic contributions [17,18]. The purpose of this experiment is therefore not only to measure whether larger phase fluctuations lower the fidelity, but also to determine how the relation between the segment duration $\tau_{c}$ and the characteristic temporal scales selected by the CPMG pulse pattern affects the circuit response.

**Figure 2:**
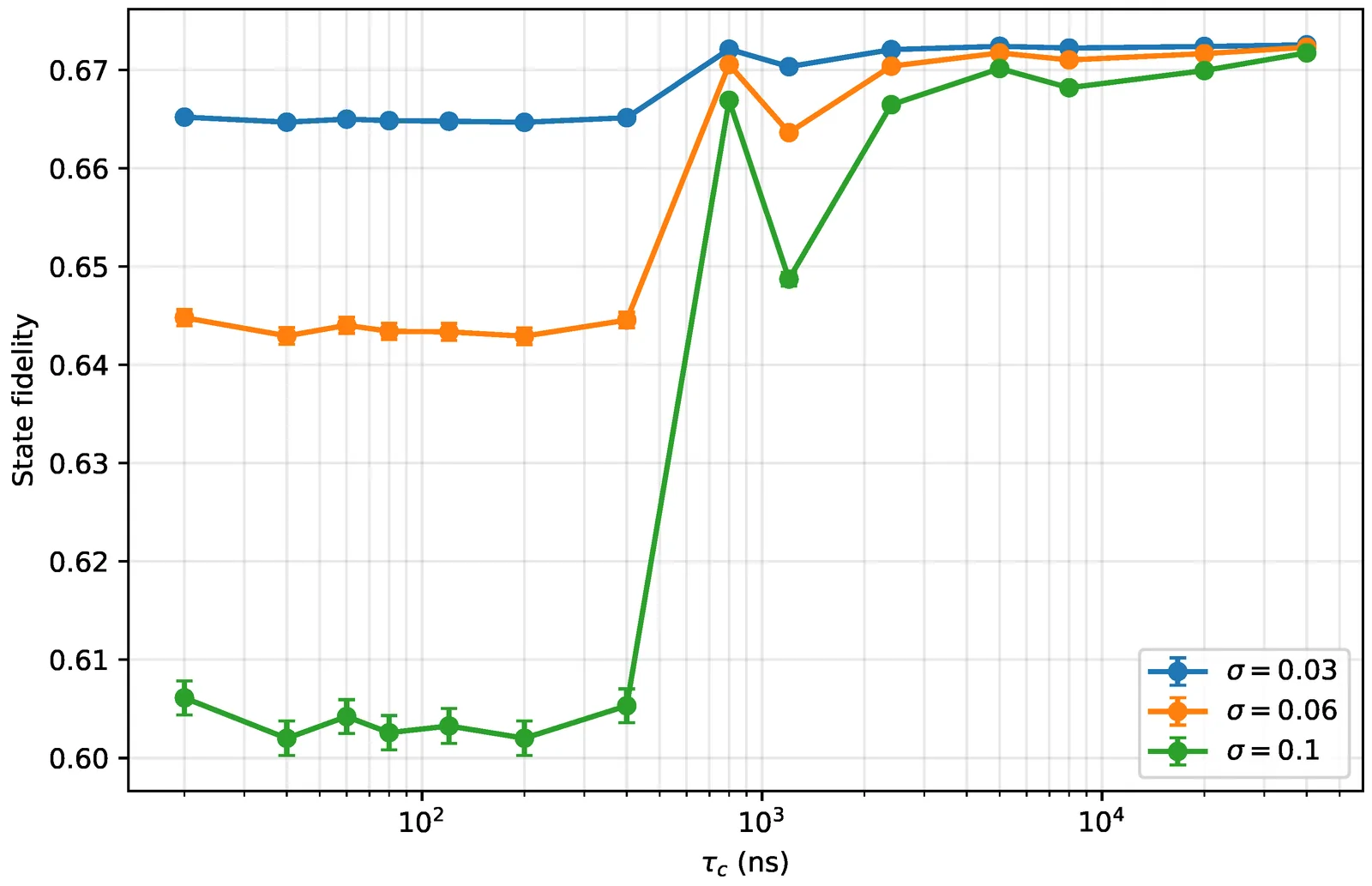
CPMG output-state fidelity under segmented flux-induced phase noise at different fluctuation strengths. The circuit-output fidelity of the single-qubit CPMG sequence as a function of the model correlation-time parameter $\tau_{c}$ for three fluctuation strengths, σ=0.03 , 0.06 , and 0.10 , under the common composite-noise background. The circuit contains 100 refocusing pulses and a waiting duration of 400 ns between pulses. The horizontal axis is shown on a logarithmic scale. Each marker shows the sample mean of the standard output-state fidelity over 2000 independently sampled noisy realizations, and the error bars indicate the standard error of the mean. Where not visible, the error bars are smaller than the plotted markers.

Figure 2 shows a clear transition between the short- and long- $\tau_{c}$ regimes. Using the standard output-state fidelity, as $\tau_{c}$ increases from the shortest tested value of 20 ns to the longest tested value of 40,000 ns, the mean fidelity increases by approximately $7.4\times 10^{-3}$ , $2.8\times 10^{-2}$ , and $6.6\times 10^{-2}$ for σ=0.03 , 0.06 , and 0.10 , respectively. These changes correspond to approximately 0.74 , 2.8 , and 6.6 percentage points.

To assess whether these changes could arise from the finite number of sampled noise realizations, we also estimate the uncertainty of each difference using the independent-mean-difference standard error defined in Section 3.3. The corresponding standard errors of the endpoint differences are approximately $2.3\times 10^{-4}$ , $8.2\times 10^{-4}$ , and $1.7\times 10^{-3}$ for σ=0.03 , 0.06 , and 0.10 , respectively. In every case, the observed fidelity increase is much larger than its estimated finite-sample uncertainty. The transition with $\tau_{c}$ is therefore clearly resolved relative to fluctuations in the Monte Carlo estimates caused by the finite ensemble size. Across the three tested strengths, the absolute fidelity changes span approximately 0.74 – 6.6 percentage points, directly quantifying the scale of the $\tau_{c}$ dependence at the single-qubit circuit level.

The dependence on the fluctuation strength also becomes much weaker in the long- $\tau_{c}$ regime. At $\tau_{c}=20$ ns, the fidelity difference between σ=0.03 and σ=0.10 is approximately $5.9\times 10^{-2}$ , whereas at $\tau_{c}$ = 40,000 ns it is approximately $8.1\times 10^{-4}$ , representing a reduction of approximately 98.6% . The three curves therefore approach a narrow plateau near an output-state fidelity of 0.672 , although they do not become exactly identical. This behavior is consistent with the interpretation that, when the segment-wise phase offsets vary slowly relative to the CPMG pulse pattern, the sequence refocuses a larger fraction of the accumulated phase perturbation and becomes substantially less sensitive to the fluctuation strength. The local non-monotonic adjacent changes in Figure 2 are retained in the plotted data, but the interpretation relies on the endpoint increase, the compression of the σ -dependence, and the boundary-assignment robustness check rather than on pointwise monotonicity.

The boundary-assignment robustness check gives the same qualitative conclusion. Under the fixed segment origin, replacing the maximum-overlap rule by start time, midpoint, or overlap-weighted random assignment leaves the CPMG trend essentially unchanged for the tested setting. Randomizing the segment origin produces only small pointwise shifts and preserves the endpoint increase and the approach to the large- $\tau_{c}$ plateau. These checks indicate that the transition observed in Figure 2 is not a consequence of a particular deterministic segment boundary or of the maximum-overlap assignment convention.

Since the non-flux background channels are identical for all values of $\tau_{c}$ , they set a common baseline for the comparison rather than introducing an additional $\tau_{c}$ -dependent control parameter. The $\tau_{c}$ -dependent changes in Figure 2 are therefore interpreted in terms of the segmented temporal organization of the flux-induced phase offsets under this fixed composite-noise background.

### 4.2. Depth-Dependent $\tau_{c}$ Response in Randomized Benchmarking

We next turn to standard single-qubit randomized benchmarking and examine how the mean survival probability at a fixed circuit depth changes with the model parameter $\tau_{c}$ . In this experiment, the fluctuation strength is fixed at σ=0.10 , and the mean survival is reported for three RB depths: m=64 , 256, and 1024. The plotted error bars denote the standard error of the mean over 300 independently sampled sequence–noise pairs. The purpose of this figure is not to analyze the detailed RB decay shape, but to determine how the effective role of $\tau_{c}$ depends on the time scale of the circuit itself.

**Figure 3:**
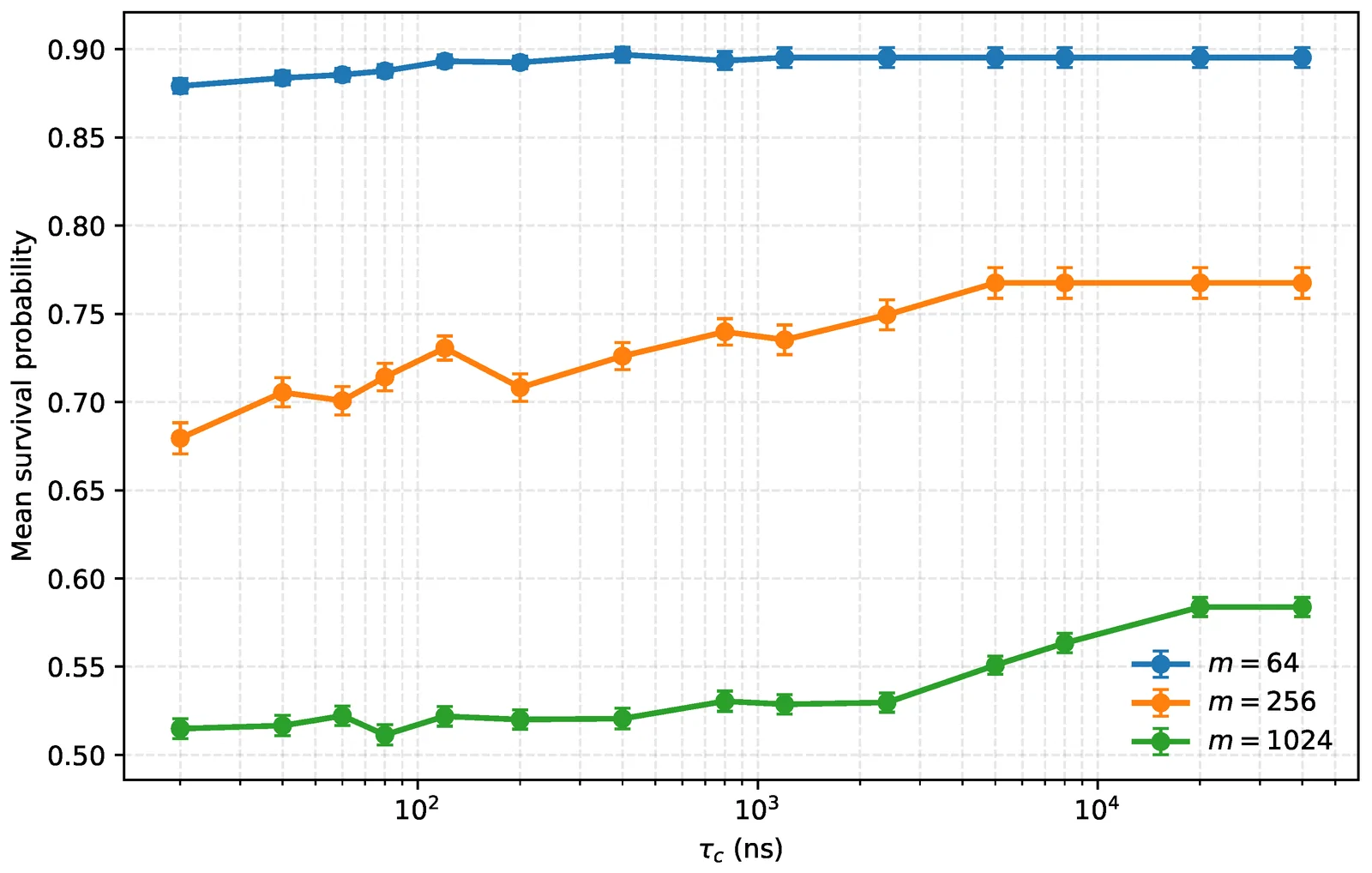
Depth-dependent $\tau_{c}$ response in randomized benchmarking. Mean survival probability of standard single-qubit randomized benchmarking as a function of the model correlation-time parameter $\tau_{c}$ at three circuit depths, m=64 , 256, and 1024, with the fluctuation strength fixed at σ=0.10 . Each marker shows the sample mean over 300 independently sampled sequence–noise pairs, and error bars indicate the standard error of the mean.

Figure 3 shows a clear depth dependence. For the shallowest circuit, m=64 , the mean survival changes only moderately across the full $\tau_{c}$ range and reaches a stable high- $\tau_{c}$ plateau relatively early. For the intermediate depth, m=256 , the $\tau_{c}$ -dependence becomes much more pronounced and exhibits the largest visible dynamic range in the present data. For the deepest circuit, m=1024 , the overall variation is not the largest, but the onset of the high- $\tau_{c}$ plateau is shifted substantially to the right.

To interpret this observed rightward shift, we reconstruct the execution durations of the sampled RB circuits. The maximum durations among the 300 sampled sequences are approximately 3.14μs , 11.38μs , and 44.40μs for m=64 , 256, and 1024, respectively. These values increase with the RB depth and provide an execution-time explanation for why the high- $\tau_{c}$ plateau appears later for deeper circuits. In particular, the tested values $\tau_{c}=5000$ ns and $\tau_{c}$ = 20,000 ns already exceed the maximum sampled durations for m=64 and m=256 , respectively, whereas the largest tested value $\tau_{c}$ = 40,000 ns remains slightly below the maximum sampled duration for m=1024 . This comparison supports the interpretation that the apparent plateau onset is tied to how the circuit-execution duration compares with the model correlation-time parameter $\tau_{c}$ .

This execution-time comparison clarifies the observed rightward shift in the plateau onset. It shows that the value of $\tau_{c}$ at which the segmented phase process appears effectively slow is not universal, but depends on the circuit time scale. A shallow RB circuit spans a shorter execution window and therefore reaches its high- $\tau_{c}$ plateau at a comparatively smaller $\tau_{c}$ . By contrast, a deeper circuit extends over a much longer execution time, so the same nominal $\tau_{c}$ may still correspond to multiple noise segments within a single circuit execution. Consequently, a larger $\tau_{c}$ is required before the segmented phase process appears effectively slow over the full circuit duration.

The depth dependence in Figure 3 is therefore best understood in terms of two complementary effects. Intermediate-depth circuits, represented here by m=256 , display the largest visible response amplitude over the scanned $\tau_{c}$ range. Deeper circuits, represented here by m=1024 , more clearly reveal the rightward shift in the onset of the high- $\tau_{c}$ plateau. Taken together, these results show that the role of the segmented flux-induced phase process depends on how the circuit duration compares with $\tau_{c}$ : different depths emphasize different aspects of the same underlying $\tau_{c}$ dependence. The local non-monotonic adjacent changes in Figure 3 are retained in the plotted data rather than being smoothed. These local decreases are isolated and are not used as separate physical trend claims. The main interpretation of Figure 3 instead relies on the depth-dependent shift in the apparent plateau onset, the comparison with reconstructed circuit durations, and the overall dependence of the survival response on how the circuit-execution duration compares with $\tau_{c}$ .

### 4.3. RB Decay Profile Under Segmented Flux-Induced Phase Noise

We next examine the full survival-decay curves of standard single-qubit randomized benchmarking in order to determine how the temporal structure of the segmented phase process modifies the shape of the RB response. In this experiment, the fluctuation strength is fixed, and $\tau_{c}$ is the only scanned parameter. The purpose is therefore to isolate how varying $\tau_{c}$ reshapes the survival decay across sequence depth under otherwise fixed noise magnitude.

**Figure 4:**
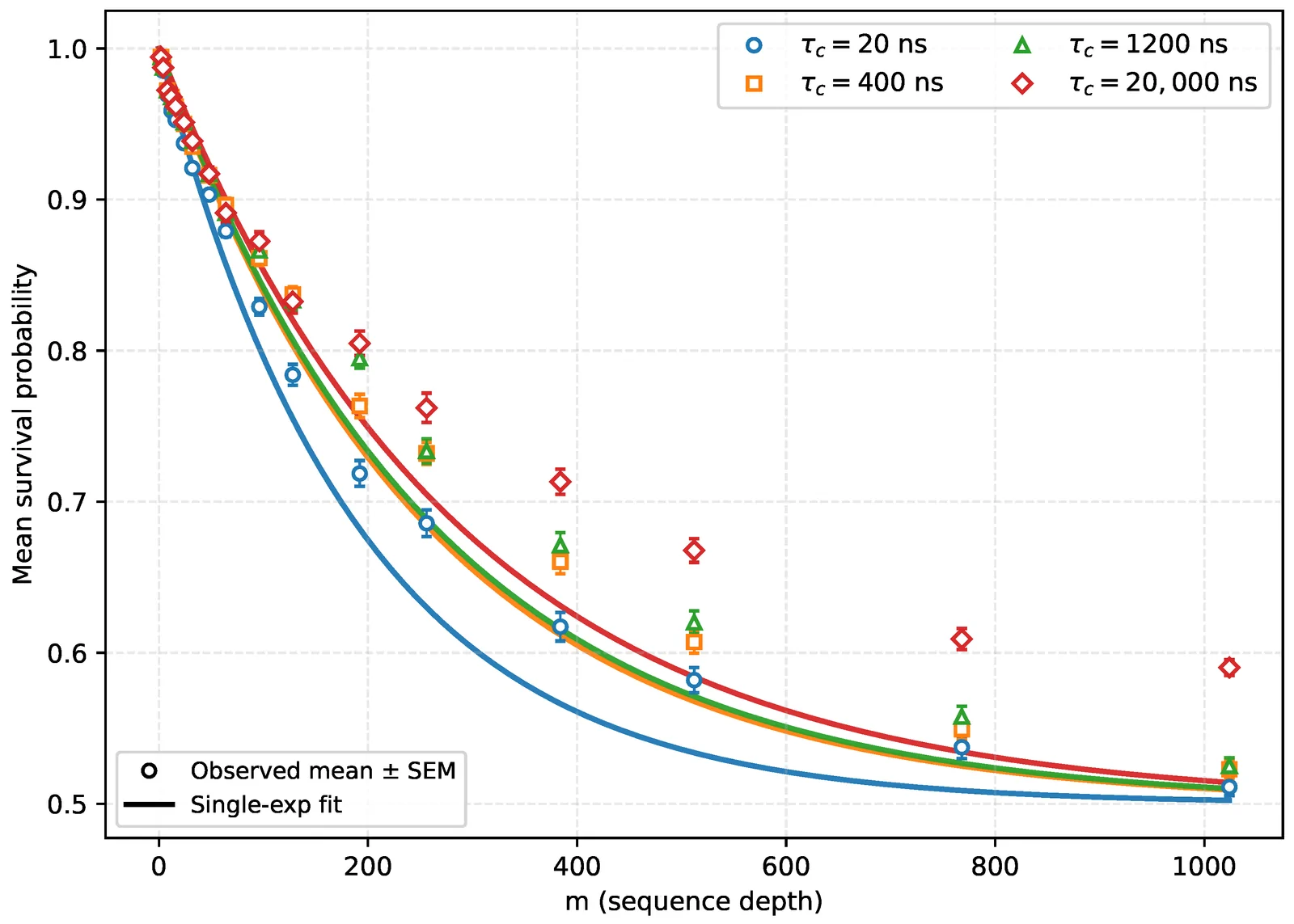
RB decay shape under segmented flux-induced phase noise at representative values of model correlation-time parameter. Mean survival probability of standard single-qubit randomized benchmarking as function of sequence depth *m* for four representative values of $\tau_{c}$ , $\tau_{c}=20$ , 400, 1200, and 20,000 ns, with fluctuation strength fixed at σ=0.10 . Markers show sample mean over 300 independently sampled sequence–noise pairs, error bars indicate standard error of mean, and solid curves show corresponding single-exponential reference fits.

Figure 4 shows that the RB decay becomes progressively less well described by a single-exponential reference form as the model correlation-time parameter $\tau_{c}$ increases [21,22]. The single-exponential curve is used here as a reference fit for standard RB decay behavior, rather than as an assumption that the temporally correlated data must be exactly exponential. For the short-correlation case $\tau_{c}=20$ ns, the observed data remain close to the fitted single-exponential curve over the full depth range. At $\tau_{c}=400$ ns, the agreement is still reasonably good, although small deviations begin to appear at intermediate depths. For $\tau_{c}=1200$ ns, the mismatch becomes more visible, especially once the sequence depth enters the intermediate-to-large range. This trend reflects the temporal-memory effects represented by the segmented phase-noise construction at the circuit level. As $\tau_{c}$ increases, the sampled phase perturbation remains correlated and varies more slowly over larger portions of a given RB sequence, so the phase bias experienced within each realization becomes more strongly correlated across successive parts of the execution. The ensemble of such temporally correlated realizations produces a survival decay that is no longer well represented by a single exponential, and the deviation becomes increasingly visible at intermediate and large sequence depths.

The clearest departure appears in the long-correlation case $\tau_{c}$ = 20,000 ns. In this regime, the observed survival probabilities remain systematically above the corresponding single-exponential fit at intermediate and large depths, producing a visibly elevated tail. Increasing $\tau_{c}$ therefore reshapes the RB decay itself, with the late-depth portion becoming progressively slower relative to the standard single-exponential reference.

Figure 4 thus shows that the segmented flux-induced phase-noise process produces a progressive distortion of the RB decay profile, and that this distortion becomes most apparent in the middle and late portions of the sequence. In this regime, the RB response is characterized not only by an effective fitted decay parameter, but also by a visible change in the overall decay shape.

### 4.4. Quantifying the Deviation from the Single-Exponential RB Picture

To summarize the trend visible in Figure 4, we extract from the same RB survival data the fitted single-exponential decay parameter *p* together with two fit-quality diagnostics, namely the root-mean-square error (RMSE) and the maximum absolute residual. These quantities are used to compare the RB survival curves with a single-exponential reference form.

**Figure 5:**
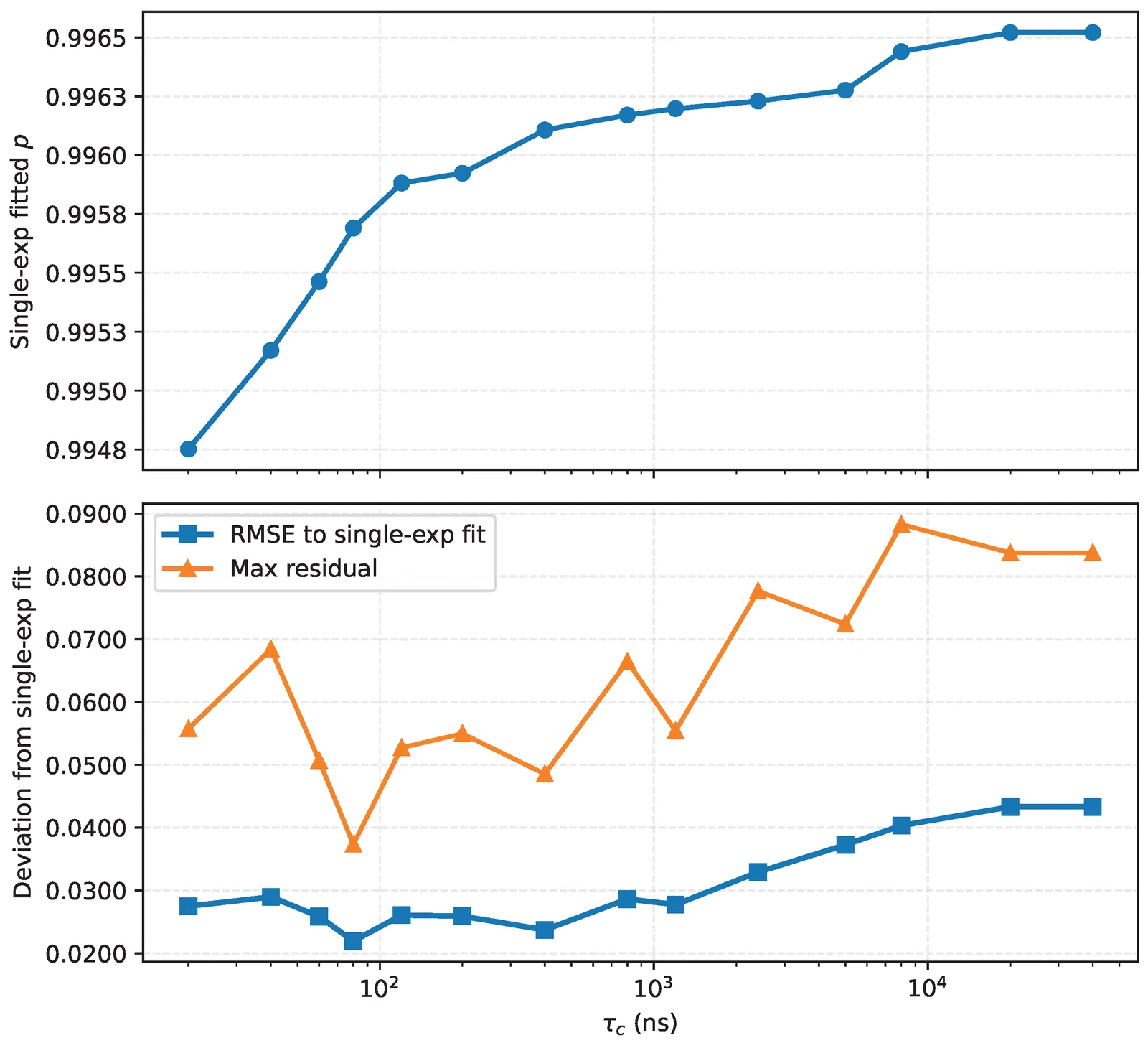
Single-exponential RB fit diagnostics under temporally correlated flux-induced phase noise. The upper panel shows the fitted decay parameter *p* as a function of the model correlation-time parameter $\tau_{c}$ . The lower panel shows the corresponding root-mean-square error and maximum absolute residual relative to the single-exponential reference fit. All quantities are extracted from the same RB survival data under the fixed composite-noise background. Bootstrap confidence intervals for the fitted and residual-based quantities are computed from the same sequence–noise pairs and reported in the text.

Figure 5 shows that the fitted parameter *p* varies only within a relatively narrow range over the full $\tau_{c}$ sweep. By contrast, both the RMSE and the maximum absolute residual increase substantially as $\tau_{c}$ enters the long-correlation regime and reach their largest values at the high- $\tau_{c}$ end of the sweep. This indicates that the effect of increasing $\tau_{c}$ is expressed more clearly in the shape of the RB survival curve than in the fitted decay parameter alone.

Quantitatively, the fitted EPC decreases from $2.62\times 10^{-3}$ at $\tau_{c}=20\;\mathrm{ns}$ to $1.74\times 10^{-3}$ at $\tau_{c}$ = 40,000 ns, corresponding to the increase in the fitted *p* shown in the upper panel of Figure 5. The bootstrap 95% confidence intervals for the EPC are $\left[2.54,2.70\right]\times 10^{-3}$ and $\left[1.61,1.89\right]\times 10^{-3}$ , respectively. Over the same range, the RMSE of the single-exponential reference fit increases from $2.75\times 10^{-2}$ to $4.33\times 10^{-2}$ , with bootstrap 95% confidence intervals $\left[2.43,3.11\right]\times 10^{-2}$ and $\left[3.93,4.95\right]\times 10^{-2}$ . The maximum absolute residual similarly increases from $5.57\times 10^{-2}$ to $8.38\times 10^{-2}$ , with bootstrap 95% confidence intervals $\left[4.74,7.31\right]\times 10^{-2}$ and $\left[7.67,10.21\right]\times 10^{-2}$ .

The representative nested diagnostic further separates the two sources of finite-sample variation. In the selected settings, noise-realization variation is the dominant component, accounting for approximately 75– 97% of the estimated two-component variation. The sequence-to-sequence component is non-negligible but secondary, contributing approximately 3– 25% , and becomes more visible at larger $\tau_{c}$ . This supports interpreting the main RB uncertainty as a joint sequence–noise uncertainty, with the noise-realization component providing the larger contribution in the diagnostic settings.

These results show that the dominant effect of increasing $\tau_{c}$ is expressed more clearly in fit quality than in the fitted decay parameter itself. As the sampled phase perturbation becomes more slowly varying over a circuit run, the RB survival curve departs progressively from the single-exponential reference form, even though the corresponding best-fit value of *p* changes only moderately. In this sense, the fitted parameter remains a useful effective reference quantity, while the residual-based diagnostics provide a more informative summary of the shape distortion induced by temporally correlated flux-induced phase noise. We emphasize that these residual-based diagnostics quantify departures from the single-exponential RB reference fit; they are not used as a formal test of quantum non-Markovianity in the open-system sense.

## 5. Conclusions and Outlook

In this work, we developed an execution-oriented, circuit-level workflow for modeling and evaluating temporally correlated flux-induced phase noise in superconducting quantum circuits. The workflow combines source-specific circuit-level noise components with a segmented correlation-time construction, allowing the associated correlation-time parameter $\tau_{c}$ to be varied under otherwise matched simulation conditions. This design makes it possible to study how temporally structured flux-induced phase noise is expressed at the level of circuit execution rather than only through isolated component metrics or gate-averaged descriptions.

Our results show that temporally correlated flux-induced phase noise produces circuit-level behavior that differs qualitatively from an uncorrelated or memoryless noise description. Its impact is governed jointly by the correlation-time parameter $\tau_{c}$ , the temporal structure of the circuit, and the way in which the circuit samples the noise, as reflected in both the CPMG fidelity response and the depth- and shape-dependent behavior of randomized benchmarking.

This study is limited to circuit-level modeling and numerical evaluation. The present construction is intended as a phenomenological, execution-level representation for controlled analysis of temporal-memory effects in circuit execution. Its present role is to support controlled comparisons of how a temporal-memory structure interacts with pulse timing and circuit duration. It is complementary to device-calibrated spectral descriptions and microscopic open-system models. Device-specific quantitative prediction would require calibration of the execution-level covariance to measured noise spectra or time-domain records, together with the corresponding operating-point sensitivities and control functions. In addition, the present evaluation focuses on single-qubit CPMG and randomized benchmarking circuits, which provide two complementary testbeds for resolving the circuit-level role of $\tau_{c}$ . Multiqubit extensions can build on this basis to incorporate correlated errors, entangling-gate dynamics, leakage, spectator effects, syndrome-extraction cycles, spatial correlations, and decoder- and logical-level behavior.

Several directions follow naturally from this work. One is to extend the same segmented correlation-time framework to multiqubit circuits in which entangling gates and execution scheduling play a more dominant role. Another is to strengthen the connection between the present circuit-level description and frequency-domain interpretations, so that the relationship among pulse structure, effective filter characteristics, and noise statistics can be analyzed more systematically. A concrete step in this direction is to compare the segmented execution-level representation with calibrated 1/f -type, Ornstein–Uhlenbeck, and experimentally reconstructed noise processes under explicitly matched phase-variance, covariance, or circuit-response criteria. It will also be useful to examine how temporally correlated flux-induced phase noise affects the interpretation of benchmarking results and whether similar effects persist in more application-oriented circuits, compilation strategies, and error-mitigation or error-correction settings. More broadly, these directions may help to connect temporally correlated noise modeling more directly to performance analysis and design questions in superconducting quantum computing.

## Figures and Tables

**Table 1:** Common timing and noise parameters used in the evaluation.

Parameter	Value
**Timing**	
Single-qubit gate duration $t_{1}$	20 ns
Two-qubit gate duration $t_{2}$	40 ns
**Idle noise**	
$T_{1}$	30 μ s
$T_{\phi }$	60 μ s
**Single-qubit control noise**	
$p_{\mathrm{axis}}$	$10^{-4}$
$p_{\mathrm{plane}}$	$5\times 10^{-4}$
**Photon-induced dephasing**	
χ/π	−2.6 MHz
$\alpha_{0}$	$\sqrt{0.8}$
κ	$4\times 10^{6}\;s^{-1}$
$\tau_{m}$	−590 ns
$\tau_{g}$	10 ns

## Data Availability

The code and configuration files used in this study are publicly available at https://github.com/diaodaliuhe/cirq_project (accessed on 12 December 2025).
